# Research progress in medical imaging for intracranial aneurysms: technological advances in diagnosis, management, and clinical integration

**DOI:** 10.3389/fneur.2025.1657536

**Published:** 2025-08-28

**Authors:** Yuwei Zhou, Wei Weng

**Affiliations:** ^1^Zhejiang Chinese Medical University, Hangzhou, Zhejiang, China; ^2^Department of Radiology, Wenzhou People’s Hospital, Wenzhou, Zhejiang, China; ^3^Department of Radiology, The Third Affiliated Hospital of Wenzhou Medical University, Wenzhou, Zhejiang, China

**Keywords:** intracranial aneurysms, medical imaging, computed tomography angiography, magnetic resonance angiography, digital subtraction angiography, artificial intelligence, deep learning, cerebral hemodynamics

## Abstract

Intracranial aneurysms (IAs) represent a significant cerebrovascular disorder that has attracted considerable scrutiny due to the elevated rates of mortality and morbidity associated with their rupture. The ongoing evolution of medical imaging techniques has led to the emergence of non-invasive imaging options, including Computed Tomography Angiography (CTA), Magnetic Resonance Angiography (MRA), and Digital Subtraction Angiography (DSA). These modalities are essential for the early detection, risk evaluation, and therapeutic strategy formulation for IAs. Recently, the integration of artificial intelligence (AI) and three-dimensional (3D) reconstruction technologies has further improved the precision and efficiency of imaging diagnostics. This review provides a systematic assessment of advancements in imaging diagnostic methods for IAs, covering both established and novel imaging techniques, AI-enhanced diagnostics, hemodynamic evaluations, the role of imaging in treatment, and prospective development directions. The objective is to furnish thorough references for clinical diagnosis and investigation in this vital field of medicine.

## 1 Introduction

Intracranial aneurysms (IAs) are abnormal dilations of cerebral arteries that pose a significant risk of rupture, often leading to subarachnoid hemorrhage (SAH), a severe form of stroke with high morbidity and mortality (1).

Large-scale epidemiological meta-analyses estimate the prevalence of unruptured intracranial aneurysms (UIAs) in the general population at approximately 3.2% (95% CI 1.9–5.2), with higher incidence observed in women and in individuals with hypertension or a family history of aneurysms (2). The fatality rate following aneurysm rupture is estimated at around 35%, and many survivors experience lasting neurological impairments (2).

Despite the clinical importance of early diagnosis, the identification and characterization of IAs remain challenging due to the complexity of cerebrovascular anatomy and the often asymptomatic nature of unruptured aneurysms. Conventional imaging modalities—such as computed tomography angiography (CTA), magnetic resonance angiography (MRA), and digital subtraction angiography (DSA)—remain fundamental tools for aneurysm detection, risk stratification, and treatment planning (3).

This review provides a comprehensive assessment of current and emerging diagnostic strategies for IAs, with an emphasis on imaging-based techniques. We summarize the capabilities and limitations of conventional modalities and examine the role of advanced technologies—including hemodynamic modeling and AI-assisted analysis—in improving diagnostic accuracy and clinical decision-making. By integrating clinical relevance with recent research progress, this review aims to inform both medical practice and future investigative directions in aneurysm imaging.

### 1.1 Imaging foundations and diagnostic significance of IAs

This section presents an overview of conventional and advanced imaging techniques used in the diagnosis and evaluation of IAs, including AI-assisted tools, CFD modeling, and high-resolution vessel wall imaging (HR-VWI).

**Figure 1:**
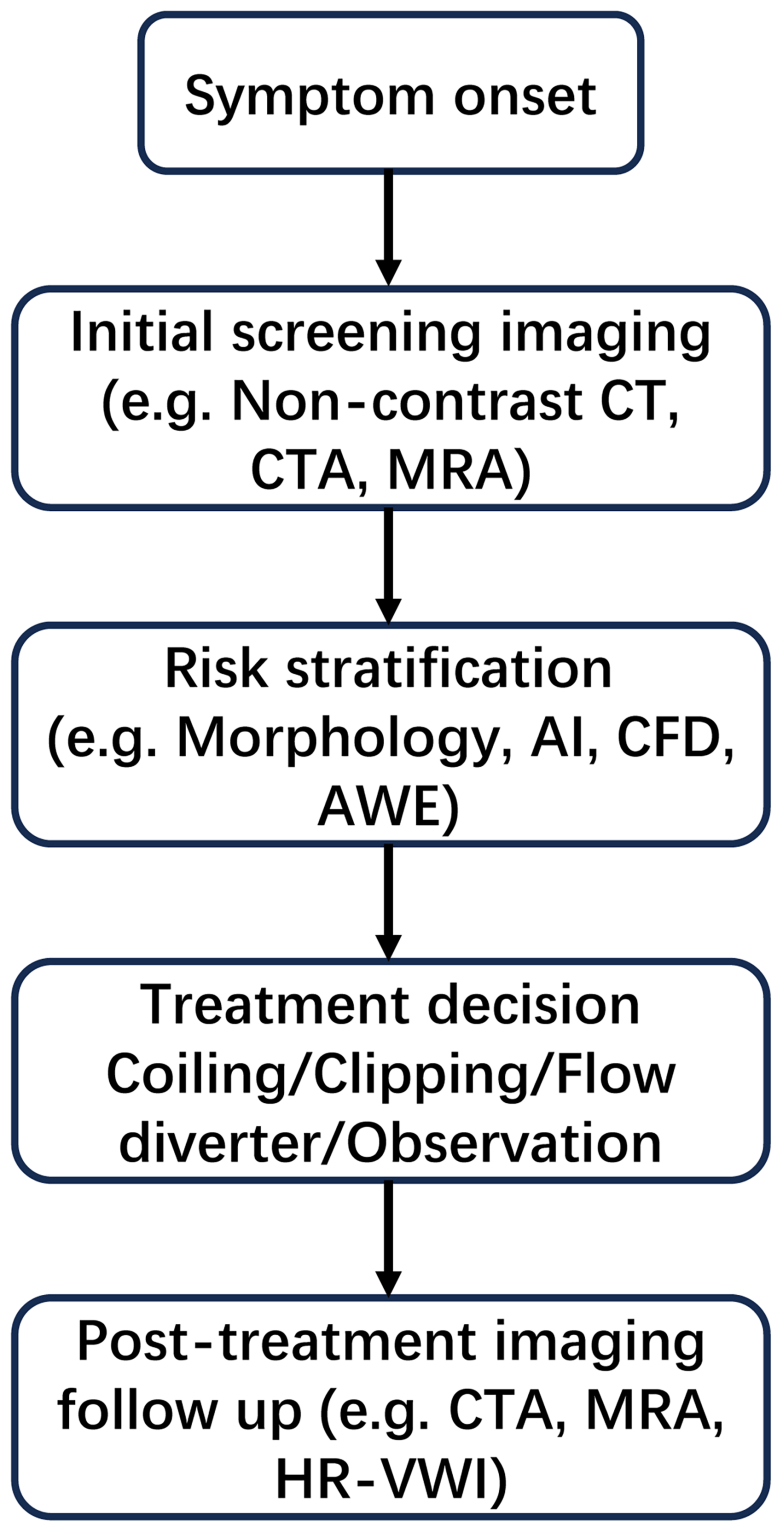
Clinical decision-making workflow.

To provide a clear overview of the clinical context in which imaging technologies are applied, Figure 1 illustrates the standard diagnostic and decision-making workflow for IAs. The process begins with symptom presentation and progresses through sequential steps including screening imaging, risk stratification based on morphological and hemodynamic parameters, treatment selection (e.g., coiling, clipping, or observation), and post-treatment imaging follow-up. This schematic serves to contextualize the subsequent sections, each of which addresses key imaging techniques and analytical tools used at different stages of this clinical pathway.

#### 1.1.1 Pathophysiology and imaging characteristics of IAs

The development of IAs is a multifaceted process shaped by an array of pathophysiological mechanisms, which encompass hemodynamic stress, genetic factors, and inflammatory responses. These aneurysms most commonly arise at arterial bifurcations and vessel curvatures, where complex flow patterns and elevated wall shear stress (WSS) contribute to local weakening of the vessel wall. Imaging is integral to the identification of these aneurysms, with techniques such as magnetic resonance imaging (MRI) and CTA offering comprehensive visualization of the aneurysm’s morphology, size, and location. MRI, particularly high-resolution imaging of the vessel wall, has proven to be an essential tool in evaluating the structural integrity of aneurysm walls, permitting the identification of wall enhancement that may signify inflammation or instability (4). Moreover, the relationship between imaging findings and the risk of rupture has become a key research focus, as certain imaging features—like wall thickness and enhancement patterns—have been linked to an elevated risk of rupture. For example, research indicates that aneurysms demonstrating substantial wall enhancement on MRI are at a heightened risk for rupture, highlighting the significance of advanced imaging techniques in risk stratification (5). Additionally, the advent of dynamic contrast-enhanced MRI (DCE-MRI) has shed light on the permeability of aneurysm walls, further contributing to risk assessments and potential management strategies (6).

#### 1.1.2 The role and significance of imaging in IAs diagnosis

Imaging modalities play a critical role in the diagnosis and management of IAs, particularly concerning early detection and the evaluation of rupture risk. The capability to visualize aneurysms non-invasively has revolutionized clinical practice, facilitating the identification of unruptured aneurysms that might otherwise remain asymptomatic. The importance of early detection is paramount, as prompt diagnosis can enable appropriate intervention strategies, potentially averting catastrophic events such as SAH. DSA is widely regarded as the gold standard for assessing aneurysm morphology, while CTA serves as a rapid, non-invasive, and widely available first-line tool, especially valuable in emergency settings; however, advancements in MRI techniques are increasingly acknowledged for their ability to yield supplementary information regarding the aneurysm wall and adjacent structures (7). Furthermore, the incorporation of AI into imaging analysis has demonstrated potential in improving diagnostic accuracy and efficiency, allowing for automated detection and characterization of aneurysms (8). The significance of imaging transcends mere diagnosis; it also aids treatment decision-making by providing critical insights into aneurysm characteristics that influence management strategies, ranging from conservative observation to surgical intervention. As our comprehension of aneurysm pathophysiology advances, the role of imaging in guiding clinical decisions is expected to expand, emphasizing the necessity for continued research and development in this domain (9).

### 1.2 Primary imaging acquisition techniques

To aid comparison, Table 1 provides a structured overview of the major imaging modalities—DSA, CTA, MRA, and HR-VWI—highlighting key features such as diagnostic sensitivity, radiation exposure, invasiveness, and clinical application scenarios discussed in this section.

**Table 1:** Comparison of common imaging modalities for intracranial aneurysms.

Modality	Sensitivity	Radiation exposure	Invasiveness	Clinical use-case
DSA (Digital Subtraction Angiography)	Highest	High (X-ray fluoroscopy)	Invasive (catheterization)	Gold standard for detailed vascular anatomy and intervention planning
CTA (Computed Tomography Angiography)	High	Moderate (ionizing radiation)	Non-invasive	Rapid evaluation, emergency screening, aneurysm detection
MRA (Magnetic Resonance Angiography)	Moderate to High	None	Non-invasive	Preferred in pediatric patients, follow-up imaging, and contrast contraindications
HR-VWI (High-Resolution Vessel Wall MRI)	Variable; primarily an adjunct tool	None	Non-invasive	Assessment of aneurysm wall pathology and rupture risk stratification

#### 1.2.1 CTA

The CTA has become an essential diagnostic modality for IAs, largely owing to its swift image acquisition and notable sensitivity in identifying vascular irregularities. The underlying principle of CTA centers on the application of contrast agents that improve the visibility of blood vessels, utilized alongside sophisticated computed tomography (CT) imaging methodologies. A key benefit of CTA is its non-invasive nature when compared to standard angiography, facilitating prompt evaluations in emergency contexts. Research indicates that CTA demonstrates a sensitivity between 85 and 98% for the detection of IAs, with specificity rates often surpassing 90% in numerous instances (10). Additionally, the creation of multicenter CTA databases has promoted the incorporation of AI into imaging data analysis, thereby enhancing the capacity to predict characteristics of aneurysms and risks of rupture. These databases act as crucial resources for training AI algorithms that can assist radiologists in recognizing subtle imaging features indicative of aneurysm instability, ultimately improving strategies for patient management (11).

#### 1.2.2 MRA

The MRA is characterized by its non-invasive approach and the absence of ionizing radiation, rendering it especially beneficial for pediatric patients and individuals with contraindications to contrast agents. The primary advantage of MRA lies in its ability to visualize blood vessels and evaluate the morphology of aneurysms without the dangers associated with ionizing radiation, which is particularly critical for vulnerable groups such as children (12). Moreover, MRA has proven effective in assessing aneurysm features, including their size and shape, which are vital for determining appropriate treatment strategies. This technique is particularly valuable for analyzing flow dynamics and wall characteristics, offering insights into the hemodynamic factors that could lead to aneurysm rupture (11). Recent advancements in high-resolution MRA techniques have further improved the visualization of small aneurysms and their hemodynamic properties, thus enhancing diagnostic precision (13).

#### 1.2.3 DSA

The DSA is regarded as the gold standard for diagnosing IAs due to its exceptional ability to generate high-resolution images of vascular structures. This technique involves subtracting pre-contrast images from post-contrast images, which allows for a clear depiction of blood vessels while reducing background noise. The diagnostic utility of DSA is particularly pronounced in its capacity to accurately evaluate the morphology of aneurysms, including their size and neck characteristics, which are crucial for treatment planning (14). Additionally, the introduction of 3D rotational angiography (3DRA) has significantly improved the visualization of intricate vascular anatomies, facilitating detailed analyses of aneurysm morphology and adjacent structures (15). However, DSA is an invasive procedure that carries certain risks, such as complications arising from catheterization and exposure to ionizing radiation. In resource-constrained settings, the availability and expense of DSA can present significant obstacles, highlighting the need for the development of alternative imaging techniques that can deliver similar diagnostic information without the associated risks (16). Notwithstanding these obstacles, DSA continues to be an essential element in the thorough assessment and treatment of IAs, especially in situations where a minimally invasive endovascular approach is intended.

#### 1.2.4 High-resolution vessel wall imaging (HR-VWI) and 4D-flow MRI

High-resolution vessel wall imaging and time-resolved 4D-flow MRI have emerged as pivotal advanced imaging modalities for IAs assessment, complementing conventional lumen-based techniques (CTA/MRA/DSA). HR-VWI enables visualization of the aneurysm wall itself and detection of aneurysm wall enhancement (AWE), which has been associated with inflammatory activity and increased risk of aneurysm instability and rupture (17, 18). AWE patterns—such as focal or circumferential enhancement—are considered important imaging biomarkers that may aid in rupture risk stratification beyond simple aneurysm morphology (19).

4D-flow MRI provides non-invasive quantification of blood flow velocity and WSS within aneurysm sacs in three spatial dimensions over time. Studies demonstrate good correlation between 4D-flow-derived hemodynamic indices and CFD simulation results, although spatial and temporal resolution limitations remain (20, 21). Relative to CFD, 4D-flow MRI typically underestimates absolute WSS and velocity magnitudes when resolution is mismatched, but shows congruent spatial distribution patterns and is more feasible for repeated clinical assessments.

Emerging studies also integrate HR-VWI and 4D-flow MRI to develop predictive models for aneurysm instability. For example, radiomic features extracted from HR-VWI images combined with machine learning approaches achieved high area under the curve (AUC) values (0.86–0.98) for rupture risk prediction (22). Other work has shown that flow instability indicators—such as directional WSS gradients derived from 4D-flow MRI—correlate with aneurysm wall motion and growth (23).

In summary, HR-VWI and 4D-flow MRI are increasingly recognized as essential tools for evaluating aneurysm wall biology and flow dynamics, offering non-invasive biomarkers for risk stratification and therapeutic planning. Their inclusion enhances the comprehensiveness and clinical relevance of this review.

### 1.3 Image-based modeling and post-processing techniques

In recent years, advances in imaging technology have expanded beyond conventional CTA, MRA, and DSA to include high-resolution 3D reconstruction, dynamic flow modeling, and vessel wall characterization. These modalities provide a deeper understanding of aneurysm morphology, hemodynamics, and tissue pathology. This section is organized into two thematic parts: one focusing on simulation-based visualization and educational applications, and the other on hemodynamic modeling and structural vessel wall imaging (VWI).

#### 1.3.1 Three-dimensional reconstruction and simulation

##### 1.3.1.1 Principles and clinical value of 3D reconstruction technology

The advent of 3D reconstruction technology has significantly transformed the visualization of intricate anatomical structures, particularly in the evaluation of IAs. Importantly, 3D reconstruction is not a standalone imaging modality but a post-processing technique applied to source images obtained from primary modalities such as CTA, MRA, or DSA. By reformatting axial image datasets into volumetric renderings, 3D reconstruction enables detailed spatial assessment of vascular anatomy and pathology.

Common reconstruction techniques include volume rendering (VR), which generates photorealistic 3D depictions of vascular structures, and multiplanar volume reconstruction (MPVR), which displays multiple orthogonal views (e.g., axial, coronal, sagittal) derived from volumetric datasets. These methods facilitate improved identification of aneurysm morphology, dome-to-neck ratios, and their spatial orientation relative to branching vessels—critical factors in risk stratification and preoperative planning (24).

In clinical practice, semi-automated segmentation techniques such as thresholding, region-growing, or level-set algorithms are implemented using dedicated platforms such as Mimics (Materialise), ITK-SNAP, or 3D Slicer. The voxel resolution of the reconstructed volume typically ranges from 0.2 to 0.6 mm, depending on the modality and scanner configuration. This resolution directly influences the fidelity of aneurysm neck delineation and volumetric assessment. Geometric inaccuracies in the reconstruction may introduce substantial errors in defining flow domains, thereby affecting downstream simulations such as computational fluid dynamics (CFD). For example, variations in segmentation resolution and vessel surface smoothing can lead to over 20% differences in WSS values computed in CFD simulations (25).

Beyond diagnostic visualization, 3D reconstruction also contributes to preoperative planning and intraoperative navigation by enhancing clinicians’ spatial understanding of aneurysm anatomy. Studies have demonstrated that accurate 3D modeling facilitates surgical simulation and intervention planning, ultimately helping reduce intraoperative complications and improve patient outcomes (26, 27).

##### 1.3.1.2 Applications of VR technology in medical education and diagnosis

Virtual reality (VR) systems have been used in neurosurgical simulation to enhance spatial understanding of aneurysm morphology and its relation to adjacent structures. While primarily applied in educational settings, VR can also support preoperative planning by integrating 3D imaging data into immersive platforms (28).

##### 1.3.1.3 3D vascular model construction and its educational significance

The generation of 3D vascular models from imaging data supports both clinical understanding and educational utility in IAs management. These models transform 2D imaging into intuitive 3D structures, facilitating visualization of aneurysm morphology and its spatial relationship to surrounding vessels—features that are crucial for surgical planning. While not directly contributing to diagnosis, 3D models provide a supplementary tool for anatomical teaching and preoperative familiarization, particularly in training environments where access to real patient cases may be limited (29–31).

#### 1.3.2 Hemodynamic analysis and VWI

##### 1.3.2.1 Overview of CFD technology and its function in aneurysm progression

Accurate CFD modeling requires patient-specific input geometries, which are typically derived from segmented 3D imaging data as described in Section 2.3.1.1. Inlet and outlet boundary conditions are commonly modeled using pulsatile flow velocity profiles from phase-contrast MRI or literature-derived flow rates, often assuming parabolic or flat inlet profiles. Wall conditions are usually set to rigid and no-slip, although recent models have begun incorporating compliant wall dynamics. Blood is generally treated as a Newtonian fluid, although non-Newtonian models may be employed for slow-flow regions. Computational solvers such as ANSYS Fluent, OpenFOAM, or in-house finite volume codes are used for Navier–Stokes equation solving, with mesh sizes ranging from 0.1–0.3 mm and time steps of 0.001–0.01 s. Mesh independence testing and time-averaged vs. instantaneous WSS metrics are critical for result reproducibility. These modeling parameters substantially affect key output variables like WSS, oscillatory shear index (OSI), and flow residence time (32).

The CFD technology employs mathematical models to simulate fluid dynamics and assess the forces acting on vascular structures. In the context of IAs, CFD can deliver comprehensive insights into the hemodynamic conditions within the aneurysm sac, including flow dynamics and pressure distributions (33). This information is critical for comprehending the mechanisms that contribute to aneurysm formation and rupture. For instance, studies have revealed that irregular flow patterns, such as the presence of vortices or stagnation points, can trigger endothelial cell activation and inflammatory responses, potentially facilitating aneurysm growth. By utilizing patient-specific data obtained from imaging techniques, CFD can generate highly precise models that reflect the distinct anatomical and hemodynamic features of individual patients. This personalized approach to hemodynamic assessment not only deepens our comprehension of aneurysm pathology but also lays the groundwork for customized treatment plans that consider each patient’s specific risk profile (34, 35). CFD analysis provides multiple quantitative indices relevant to aneurysm wall pathology. Table 2 summarizes key hemodynamic parameters derived from CFD models and their clinical implications.

**Table 2:** Common hemodynamic parameters derived from CFD and their clinical interpretation in intracranial aneurysms.

CFD Indicator	Definition/measurement	Clinical significance
Wall shear stress (WSS)	Frictional force exerted by blood flow tangential to vessel wall	Low WSS linked to endothelial dysfunction and aneurysm growth; High WSS may contribute to rupture at focal stress points
Oscillatory shear index (OSI)	Quantifies change in WSS direction over cardiac cycle	High OSI indicates disturbed flow and correlates with inflammation, wall remodeling, and rupture risk
Relative residence time (RRT)	Inverse of WSS adjusted by OSI; reflects flow stagnation	Elevated RRT suggests prolonged particle exposure to wall, promoting thrombus and remodeling
Flow velocity/inflow jet	Speed and direction of blood entering aneurysm sac	High-velocity inflow jets can impinge on the dome, increasing local stress; associated with unstable flow patterns
Vorticity/flow complexity	Qualitative or quantitative assessment of intra-aneurysmal vortex structures	Highly complex/vortical flow linked to aneurysm instability and rupture-prone behavior
Pressure distribution	Local pressure within the aneurysm sac or wall	Localized high pressure may correspond to areas of wall thinning or rupture sites

##### 1.3.2.2 Hemodynamic assessment (CFD) and aneurysm risk evaluation

The CFD modeling of IAs relies on accurate three-dimensional reconstruction of vascular geometry, which is typically obtained through segmentation of imaging data such as CTA or MRA. The quality and precision of the segmented aneurysm surface play a fundamental role in defining boundary conditions and flow domains. Without anatomically realistic segmentation, CFD simulations cannot reliably compute intra-aneurysmal flow dynamics, WSS, or OSI—all of which are key predictors of aneurysm rupture. Therefore, segmentation serves as the essential bridge between medical imaging and quantitative hemodynamic analysis (25, 32).

Computational fluid dynamics signifies a notable progression in evaluating hemodynamic factors related to IAs (32). This technology facilitates the simulation of blood flow within aneurysms, yielding insights into the hemodynamic conditions that contribute to the formation, growth, and rupture of aneurysms. By examining factors such as WSS, flow velocity, and OSI, medical professionals can gain a deeper understanding of the mechanical forces affecting the aneurysm wall. This understanding is vital for risk stratification, as specific hemodynamic profiles have been linked to an elevated risk of rupture. For example, research has indicated that areas of low WSS are associated with aneurysm growth and instability, while regions of high WSS may signal potential rupture risk. The capacity to model these intricate interactions in a patient-specific context enhances the accuracy of risk assessments and informs clinical decisions regarding monitoring and intervention strategies (36, 37).

##### 1.3.2.3 Value of CFD parameters in predicting aneurysm rupture risk

The prognostic significance of CFD parameters in evaluating the risk of aneurysm rupture has attracted substantial interest in recent years. Numerous investigations have underscored the promise of hemodynamic variables, including WSS, OSI, and flow dynamics, as potential predictors of aneurysm stability (25). For example, studies have indicated that diminished WSS correlates with an increased likelihood of aneurysm enlargement and rupture, whereas heightened WSS may suggest areas of possible instability. By incorporating CFD analysis into standard clinical workflows, medical professionals can gain critical insights into the hemodynamic properties of specific aneurysms, facilitating more informed treatment decisions. Furthermore, the capacity to forecast rupture risk through hemodynamic metrics may improve patient stratification for monitoring protocols, ultimately enhancing outcomes for patients with IAs (34, 35).

##### 1.3.2.4 Correlation of hemodynamic parameters with clinical symptoms

Recent research indicates that particular hemodynamic parameters derived from CFD analysis may be associated with clinical symptoms experienced by patients with IAs, including headaches (38). Studies have shown that patients with aneurysms displaying certain flow characteristics, such as heightened flow velocities or abnormal shear stress distributions, may have a greater likelihood of reporting headache symptoms. This relationship highlights the necessity of integrating hemodynamic analysis into the clinical assessment of patients with suspected or confirmed aneurysms. By pinpointing hemodynamic factors that correlate with symptomatology, healthcare providers can enhance their diagnostic capabilities and potentially improve patient management approaches. Moreover, comprehending the connection between hemodynamic parameters and clinical symptoms may assist in the formulation of predictive models that evaluate the risk of aneurysm rupture based on both anatomical and functional criteria (39, 40).

A prospective analysis of 96 patients demonstrated that mean dome blood velocity and time-averaged WSS were independent predictors of headache occurrence in unruptured aneurysms, with symptom relief following aneurysm treatment (39). Furthermore, a meta-analysis of 22 studies involving 1,257 patients confirmed that reduced WSS is significantly associated with rupture risk, highlighting its value as a prognostic hemodynamic marker (40).

### 1.4 Application progress of AI in imaging diagnosis of IAs

**Figure 2:**
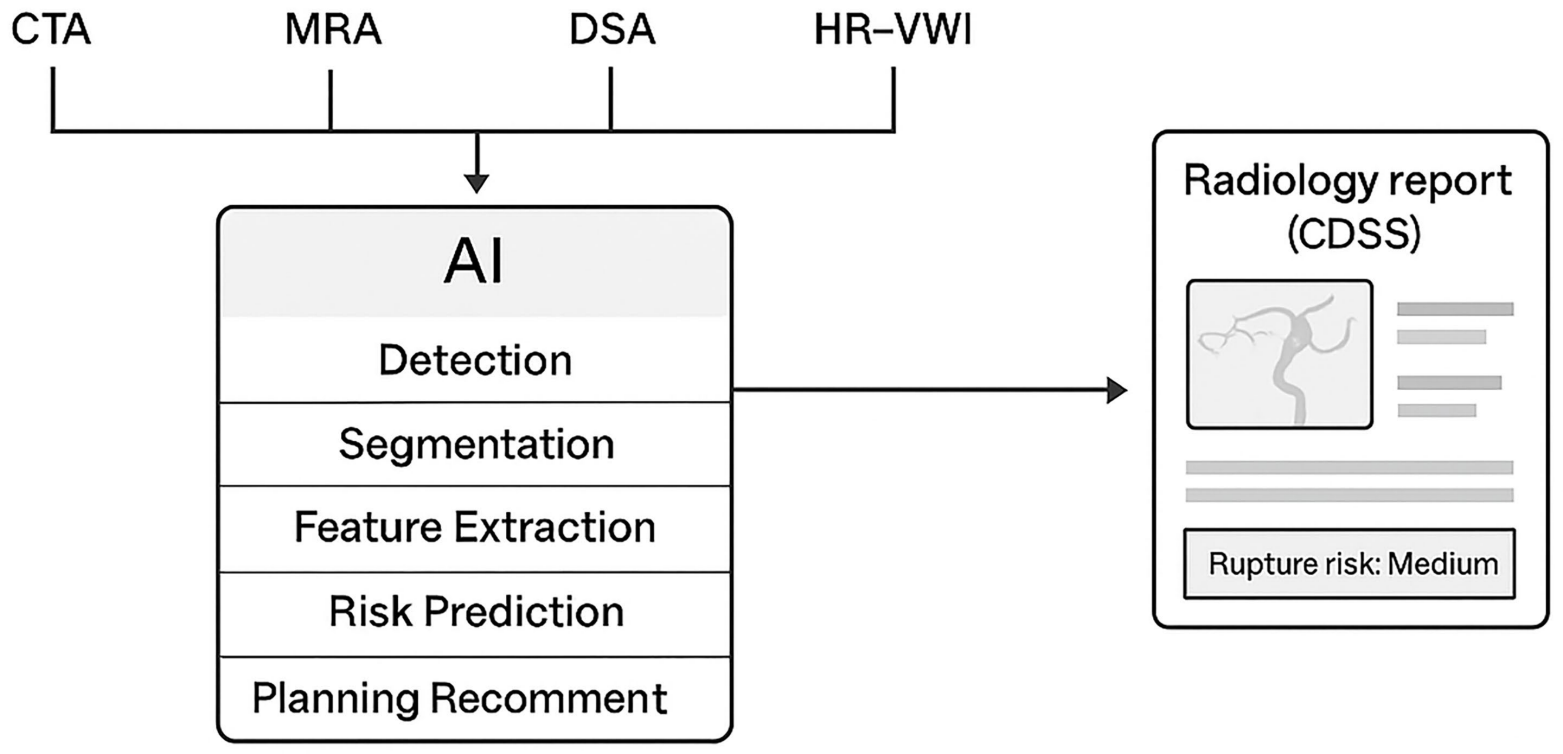
Schematic showing AI integration across modalities.

Figure 2 provides a schematic overview of how artificial intelligence (AI) is integrated across multiple imaging modalities in the diagnosis and management of IAs. AI algorithms process multimodal inputs—such as CTA, MRA, DSA, and HR-VWI—through stages including detection, segmentation, feature extraction, and risk prediction, ultimately generating outputs that support clinical decision-making and treatment planning.

With the increasing availability of large-scale imaging data and computational resources, AI has become a transformative force in neurovascular imaging. AI methods are now being applied to automate aneurysm detection, assist in segmentation, and predict rupture risk. This section discusses the latest progress in AI applications for aneurysm imaging, including technical implementations, clinical potentials, and existing challenges (41).

#### 1.4.1 AI-assisted aneurysm detection and segmentation technologies

Traditional image segmentation techniques, such as the random walk algorithm and biomechanically inspired models like the Cantilever Beam approach, have played a foundational role in the field of medical image analysis. Although initially applied to cardiac MR images for left ventricular contour extraction, these methods demonstrated robustness in delineating complex anatomical boundaries, which is also critical for accurate segmentation of IAs. For instance, early work has shown the feasibility of detecting left ventricular myocardial contours in ischemic cardiac MR using structured probabilistic models (42). Further studies have utilized random walk algorithms for LV contour segmentation (43), as well as biomechanical frameworks such as the Cantilever Beam method for multi-label segmentation of cardiac structures (44). In addition, comprehensive reviews have highlighted how data discrepancies can lead to performance divergence in classical segmentation models, emphasizing the need for more adaptive learning systems (45). These conventional techniques have laid theoretical and practical groundwork for modern AI-based approaches and remain valuable for understanding the evolution and limitations of segmentation algorithms in medical imaging.

Building on these classical techniques, deep learning models such as the 3D-Unet have been developed to automate aneurysm segmentation with improved accuracy and robustness. The 3D-Unet architecture leverages convolutional neural networks (CNNs) to enhance the precision of image analysis, allowing for voxel-wise predictions that significantly improve the identification of aneurysms compared to traditional methods. In a multicenter study, the model was trained on a substantial dataset comprising over 3,190 CTA examinations, which included 4,124 aneurysms. The performance metrics reported for this model were impressive, with a recall rate of 96.4% and a false positive rate of 2.01 per case, demonstrating its high sensitivity and specificity in detecting aneurysms. Furthermore, the Dice coefficient, which measures the overlap between the predicted segmentation and the ground truth, was reported at 0.783, indicating a robust performance in accurately delineating aneurysm boundaries (46, 47).

The efficacy of AI models in the detection of IAs is further enhanced through the utilization of multicenter databases for model training and validation. This approach not only broadens the dataset, increasing the diversity of aneurysm presentations but also mitigates the risk of overfitting, which can occur when models are trained on limited datasets. In the establishment of a multicenter database for AI applications in aneurysm imaging, data from various institutions were collated, encompassing a wide range of patient demographics and aneurysm characteristics. This comprehensive dataset allowed for the development of robust AI algorithms capable of generalizing across different populations and imaging conditions. The collaborative nature of this research also fosters the sharing of best practices and insights among institutions, leading to continuous improvements in model performance. Validation of these models against real-world clinical outcomes is crucial, as it provides evidence of their effectiveness in practical settings. The ongoing efforts to refine these AI systems through extensive training and validation processes are essential for their eventual integration into routine clinical practice, ensuring that they can reliably assist healthcare providers in the timely diagnosis and management of IAs (48).

#### 1.4.2 The potential of AI in aneurysm risk prediction and management

AI’s role in the management of IAs extends beyond detection and segmentation; it also encompasses risk prediction and treatment decision support. Automated risk scoring systems developed using machine learning algorithms can analyze various clinical and imaging parameters to stratify patients based on their rupture risk. These systems utilize a combination of morphological characteristics of the aneurysms, patient demographics, and clinical history to generate a comprehensive risk profile. For instance, AI algorithms can assess features such as aneurysm size, shape, and location, along with patient-specific factors like age and comorbidities, to predict the likelihood of aneurysm rupture. This predictive capability enables clinicians to make informed decisions regarding surveillance strategies and treatment options, potentially reducing unnecessary interventions for low-risk patients while ensuring that high-risk patients receive timely and appropriate care. Moreover, the integration of AI in clinical workflows can streamline the decision-making process, allowing for more personalized management plans that align with the individual risk profiles of patients (49, 50). A recent meta-analysis reported that deep learning models for IAs detection achieved lesion-wise sensitivity and specificity of 90 and 94%, respectively, while also improving interrater agreement among clinicians (51).

One of the significant advantages of AI in the context of IAs is its potential to minimize missed diagnoses and misdiagnoses. Traditional imaging interpretation is subject to human error, which can lead to critical delays in diagnosis and treatment. AI algorithms, trained on extensive datasets, can identify subtle patterns and anomalies in imaging studies that may be overlooked by radiologists. For instance, studies have shown that AI systems can significantly enhance the detection rates of small or atypical aneurysms, which are often challenging to diagnose accurately. By flagging these cases for further review, AI not only increases the overall diagnostic accuracy but also enhances the confidence of radiologists in their assessments. This dual approach of AI-assisted detection combined with human expertise fosters a collaborative environment where the strengths of both AI and human judgment are utilized, ultimately improving patient outcomes by ensuring that aneurysms are identified and managed appropriately (52, 53).

Recent deep learning models developed for IAs rupture risk prediction span a wide array of architectures, including two-dimensional convolutional neural networks (2D CNNs), three-dimensional U-Net (3D U-Net), no-new-Net (nnU-Net), and emerging transformer-based networks. Among these, 3D U-Net remains a dominant structure due to its encoder-decoder design with skip connections, enabling efficient voxel-wise prediction from volumetric CTA or MRA inputs. In a multicenter validation study, Yang et al. (9) developed a CNN-based model integrating both morphological and hemodynamic features, achieving an AUC of 0.92 on an external dataset. Flow dynamics and hemodynamic parameters remain critical in understanding aneurysm behavior and rupture risk. Chowdhury et al. (54) recently reviewed computational and experimental studies on flow dynamics in brain aneurysms, emphasizing the importance of integrating fluid dynamic insights with AI-based risk models to enhance predictive accuracy and clinical decision-making.

Moreover, as noted in discussions of the challenges faced, many AI-based studies in IAs imaging have been retrospective and single-center, with limited external validation, prospective evaluation, or testing in real-world clinical settings (48). These reviews collectively highlight the substantial potential of deep learning models while underscoring the need for standardized evaluation and robust clinical datasets. To enhance comparative clarity of recent AI developments in IAs imaging, Table 3 summarizes representative deep learning models, including their dataset sizes, diagnostic performance (sensitivity/specificity or AUC), and current validation or deployment stage. This structured summary provides a quick reference for understanding the technical maturity and clinical applicability of each model.

**Table 3:** Summary of major AI/deep learning models in intracranial aneurysms.

Model/Study	Dataset size	Sensitivity/specificity	Deployment stage
HeadXNet [Park et al. (47)]	818 CTA exams (611 for training, 115 for test)	Sensitivity ↑ by 5.9% with AI (from 0.799 to 0.859), Specificity no significant change	Research; clinical reader augmentation
CTA-based DL for UIA Shape/Size (96)	307 patients (training), 305 (external test); 715 UIAs total	AUC: 0.87 (shape classification); ICC: 0.92 (size measurement)	External multicenter validation
TOF-MRA DL Detection Model (97)	332 exams (135 with 168 aneurysms, 197 controls)	Sensitivity: 91.11%, Specificity: 93.91%; Lesion-wise sens: 92.26%	Preclinical; for regulatory approval in Korea

Despite promising performance, several translational barriers persist. Dataset variability—stemming from differences in scanner types, resolution, contrast timing, and labeling protocols—can significantly impair model transferability across institutions. Many studies rely on single-center data with inconsistent annotation standards and limited external validation, constraining real-world applicability (55). Moreover, while some AI systems for aneurysm detection have obtained regulatory clearance, consistent with the broader imaging-AI landscape these authorizations are limited to triage/notification or radiologist-assistive use on CTA or time-of-flight magnetic resonance angiography (TOF-MRA) rather than autonomous decision-making; consequently, rigorous regulatory compliance, explainability, and seamless clinical workflow integration remain essential for safe and effective deployment (56).

#### 1.4.3 Limitations of AI technologies and future directions

Despite the promising advancements in AI applications for IAs detection and management, several limitations persist, particularly concerning the sensitivity of AI models in detecting small aneurysms. The detection of small aneurysms, typically defined as those measuring less than 3 mm, remains a significant challenge due to their subtle imaging features and potential masking by adjacent anatomical structures. Current algorithms often exhibit reduced sensitivity for these lesions, which could lead to missed diagnoses and delayed interventions. Enhancing the detection capability for small aneurysms remains a key area for further algorithmic optimization, potentially through the integration of high-resolution imaging or hybrid multimodal data (57, 58).

Moreover, a substantial number of AI-based studies are retrospective in design and conducted in single-center settings, frequently lacking external validation, prospective evaluation, or testing in real-world clinical environments (46–48). This limitation raises concerns about the generalizability and clinical utility of the reported results. The absence of multicenter external datasets can lead to overfitting and limits the applicability of AI models across diverse patient populations and imaging protocols. To bridge the gap between experimental performance and clinical translation, future research should prioritize prospective, multicenter trials and real-world pilot deployments of AI-assisted diagnostic systems. Establishing standardized imaging protocols and unified data collection practices across institutions will be essential to ensure robust, generalizable AI models capable of supporting clinical workflows reliably (59, 60).

Furthermore, the performance of AI algorithms is heavily dependent on the quality and diversity of the training datasets. Variability in imaging acquisition parameters, such as scanner type, resolution, and contrast enhancement protocols, may affect the reproducibility and robustness of AI predictions. Therefore, cross-institutional collaboration, data harmonization efforts, and regulatory oversight will be vital to realize the full potential of AI technologies in IAs care (61, 62).

### 1.5 The role of imaging in the treatment of IAs

#### 1.5.1 Imaging-guided endovascular treatment techniques

##### 1.5.1.1 Flow diverters and their imaging characteristics

Flow diverters (FDs) have revolutionized the endovascular treatment of IAs by redirecting blood flow away from the aneurysm sac while promoting parent artery patency. These devices are typically deployed in a minimally invasive manner, and their effectiveness is largely determined by imaging techniques that visualize their placement and the subsequent hemodynamic changes. Advanced imaging modalities, such as high-resolution MRI and CTA, are essential for assessing the deployment of FDs and the immediate post-treatment changes in aneurysm morphology. Studies have demonstrated that FDs can lead to significant changes in the aneurysm sac, including reduction in size and eventual thrombosis, which can be monitored through follow-up imaging (6). Furthermore, the imaging characteristics of FDs, including the degree of contrast enhancement and the presence of intra-aneurysmal flow, provide valuable insights into the treatment’s effectiveness and the risk of complications such as aneurysm rupture or re-bleeding. The integration of these imaging modalities not only enhances the understanding of flow dynamics post-treatment but also aids in the long-term management of patients with treated aneurysms.

##### 1.5.1.2 Dual-stent-assisted coiling technique and safety assessment

The dual-stent-assisted coiling technique has emerged as a viable option for treating complex IAs, particularly those with challenging anatomical features. This method involves the placement of two stents to create a scaffold that supports the coils within the aneurysm. Imaging plays a critical role in both the procedural planning and postoperative assessment of this technique. Preoperative imaging, including DSA and CTA, is essential for evaluating the aneurysm’s morphology and the surrounding vascular anatomy, allowing for optimal stent placement (63). Postoperative imaging plays a critical role in evaluating the stability of the deployed coils and the structural integrity of the stents. Furthermore, it is essential for monitoring potential postoperative complications, including in-stent thrombosis and coil compaction, to ensure optimal patient outcomes. Safety assessments are often conducted through follow-up imaging, which can reveal complications early, allowing for timely interventions. Studies indicate that while dual-stent-assisted coiling can significantly enhance the treatment of complex aneurysms, careful imaging follow-up is necessary to ensure patient safety and treatment efficacy (64).

#### 1.5.2 Postoperative imaging follow-up and efficacy assessment

##### 1.5.2.1 Imaging indicators of aneurysm occlusion rates

Postoperative imaging is vital for assessing the efficacy of treatment strategies for IAs. The occlusion rate, defined as the percentage of aneurysms that are completely occluded post-treatment, is a key indicator of treatment success. Techniques such as DSA, CTA, and high-resolution MRI are employed to evaluate the status of the aneurysm after intervention. Studies have shown that complete occlusion rates can vary significantly depending on the treatment modality used, with endovascular coiling often yielding lower occlusion rates compared to flow diversion techniques (65). Imaging findings such as the presence of contrast filling within the aneurysm sac, changes in the aneurysm’s size, and the development of new collateral vessels are critical for determining the occlusion status. Furthermore, the use of advanced imaging techniques allows for the quantification of occlusion rates, which can be correlated with clinical outcomes, thereby providing a comprehensive understanding of the treatment’s effectiveness and guiding future management decisions.

##### 1.5.2.2 Monitoring long-term recurrence and new aneurysm formation

Long-term follow-up imaging is essential for monitoring the recurrence of treated IAs and the formation of new aneurysms. Studies indicate that patients treated for an IAs face a significant risk of developing new aneurysms or experiencing recurrences at the original site, which can occur even years after the initial treatment (66). Regular imaging follow-ups, typically using CTA or MRI, are recommended to detect these changes early. Imaging findings such as the appearance of new vascular lesions or changes in the morphology of previously treated aneurysms can indicate the need for further intervention. Moreover, advancements in imaging technology, including the use of AI for image analysis, hold promise for improving the detection of subtle changes that may precede clinical symptoms. This proactive approach to imaging follow-up not only enhances patient safety but also informs treatment strategies, ultimately improving long-term outcomes for patients with a history of IAs.

#### 1.5.3 The role of imaging in complication recognition

##### 1.5.3.1 Early diagnosis of complications such as thrombosis and hemorrhage

Imaging plays a pivotal role in the early diagnosis of complications arising from the treatment of IAs, such as thrombosis and hemorrhage. Complications can occur during or after endovascular procedures, necessitating prompt recognition to mitigate adverse outcomes. Advanced imaging modalities, including DSA and CTA, are instrumental in detecting signs of thrombosis, such as stent occlusion or coil compaction, as well as hemorrhagic events like re-bleeding from the aneurysm or surrounding vessels (48). The timely identification of these complications through imaging allows for immediate clinical intervention, which is critical in preventing significant morbidity or mortality. Furthermore, the integration of imaging findings with clinical assessments can enhance decision-making processes regarding the management of complications, ensuring that patients receive appropriate and timely care.

##### 1.5.3.2 Recognition of imaging artifacts and “phantom aneurysms”

Imaging artifacts, such as those caused by metallic devices or surrounding tissue changes, can complicate the interpretation of postoperative imaging studies for IAs. These artifacts can lead to misdiagnosis, including the phenomenon known as “phantom aneurysms,” where imaging may suggest the presence of an aneurysm that does not exist (67). Radiologists must be aware of the potential for such artifacts and develop strategies to differentiate between true aneurysms and imaging artifacts. This includes utilizing multiple imaging modalities, correlating findings with clinical data, and understanding the typical imaging characteristics of treated aneurysms. By enhancing the recognition of these artifacts, radiologists can improve diagnostic accuracy and reduce the likelihood of unnecessary interventions or anxiety for patients. The ongoing education and training of imaging specialists in recognizing these artifacts are essential to maintain high standards of care in the management of IAs.

#### 1.5.4 AI-supported imaging in treatment planning and clinical decision-making

While traditional imaging remains the foundation for IAs management, emerging technologies such as CFD and AI have begun to influence clinical decision-making. Although widespread implementation is still evolving, early case reports and technical validations suggest that these tools can enhance risk assessment and guide treatment strategies in complex scenarios.

For instance, Zhang et al. (68) described a case in which a posterior communicating artery aneurysm ruptured during angiography. CFD analysis of the rupture site revealed markedly low WSS and high OSI without evidence of flow impingement—features not typically appreciable on conventional imaging. Although urgent embolization was considered, the patient’s condition and family decision precluded intervention. The authors noted that such simulations provide insight into rupture mechanisms and may help inform treatment considerations in similar cases (68).

In another report, Toader et al. (69) described the successful microsurgical clipping of a ruptured broad-neck hypophyseal artery aneurysm in an elderly patient—anatomically unsuitable for endovascular therapy. Advanced imaging, including three-dimensional digital subtraction angiography (3D DSA) and intraoperative visualization tools, enabled precise anatomical orientation, correct clip placement, and complete aneurysm exclusion. The patient achieved full neurological recovery with no complications, and follow-up imaging at 3 months confirmed stability without recurrence. In their discussion, the authors highlighted emerging technologies such as AI-assisted rupture risk prediction and augmented reality navigation as promising adjuncts for optimizing treatment planning in complex aneurysms (69).

Beyond individual case applications, recent trials have demonstrated the potential of real-time AI systems in endovascular procedures. Masuo et al. (70) reported the first-in-human use of the Neuro-Vascular Assist platform, an intraoperative AI system designed to provide feedback during aneurysm coiling. The system analyzed microcatheter movement and coil deployment patterns in real time and issued alerts when risk thresholds were exceeded. In several cases, clinicians made immediate adjustments in response to AI guidance, potentially preventing complications. This study illustrates how AI can actively support therapeutic decision-making in dynamic procedural environments (70).

**Figure 3:**
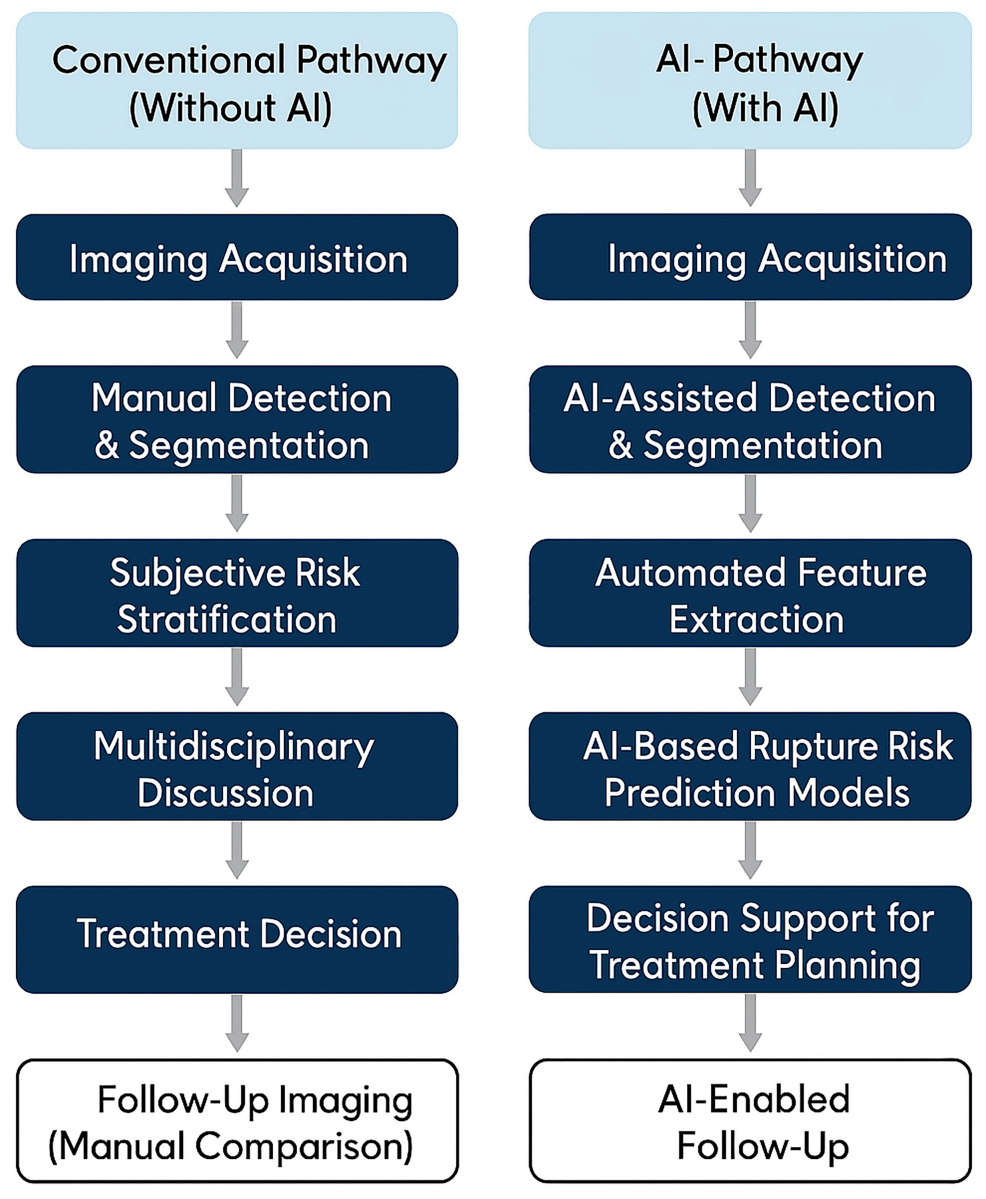
Imaging-informed management pathways with and without AI support.

To illustrate the evolving role of AI-enhanced imaging in treatment workflows, Figure 3 provides a schematic comparison of conventional and AI-augmented decision-making pathways. While standard protocols rely on visual interpretation and morphology-based judgment, AI-enabled systems incorporate automated risk prediction, CFD-based flow simulation, and procedural support tools to facilitate more personalized and potentially safer interventions.

### 1.6 Clinical challenges and misdiagnosis analysis in the imaging diagnosis of IAs

#### 1.6.1 Causes of misdiagnosis and legal risks

Misdiagnosis in the context of IAs poses significant clinical and legal challenges. One of the primary reasons for misdiagnosis is the complexity of interpreting imaging results, particularly when distinguishing between true aneurysms and vascular anatomical variants. A retrospective case series using 7 T MRI in 30 patients reported that 66% of suspected incidental aneurysms were reclassified as anatomical variants upon higher-resolution re-evaluation (71). While promising, these findings should be interpreted cautiously given the small sample size and exploratory nature of the study. Moreover, cases of delayed diagnosis can lead to severe consequences, including increased morbidity and mortality from ruptured aneurysms. The legal implications of such misdiagnoses are profound, as they often result in malpractice claims against healthcare providers. A study reviewing litigation cases related to the diagnosis and management of IAs found that failure to diagnose was the most common claim, with many cases stemming from inadequate imaging interpretation or failure to consider aneurysms in differential diagnoses (72). This highlights the need for improved training and protocols in imaging interpretation to mitigate both clinical risks and legal repercussions.

A representative case reported in that study involved a patient presenting with acute headache who underwent non-contrast CT, which was initially interpreted as negative. Due to failure to recognize subtle hemorrhagic signs and the absence of further vascular imaging, a ruptured posterior communicating artery aneurysm was diagnosed only after neurological deterioration. The delayed recognition resulted in a malpractice lawsuit and was cited as an example of inadequate escalation and radiologic misinterpretation. This case underscores the need for standardized imaging workflows, structured reporting, and enhanced training to reduce diagnostic variability and associated medico-legal risk (72).

#### 1.6.2 Diversity of clinical presentation and integration with imaging diagnosis

The clinical presentation of IAs is highly variable, which complicates the diagnostic process. Many patients present with non-specific symptoms such as headaches, which can easily be attributed to other benign conditions. For instance, a significant proportion of patients with UIAs report headaches that do not fit the classic presentation associated with ruptured aneurysms (73). This symptom overlap necessitates a careful and comprehensive diagnostic strategy that integrates clinical evaluation with advanced imaging techniques. For patients presenting without typical symptoms, such as those with only mild headaches or no neurological deficits, the challenge lies in deciding when to pursue imaging studies. Recent findings suggest that the correlation between headache symptoms and significant imaging findings is low, with many patients exhibiting normal MRI results despite chronic headache complaints (74). Therefore, establishing a robust diagnostic protocol that incorporates detailed history-taking and neurological examination may help detect indirect signs suggestive of aneurysms, particularly in atypical or symptomatic cases, thereby supporting appropriate imaging referral.

### 1.7 Imaging characteristics of IAs in children and special populations

#### 1.7.1 Clinical and imaging features of pediatric aneurysms

Pediatric IAs are notably rare, accounting for less than 5% of all IAs, and they exhibit distinct clinical and imaging characteristics compared to their adult counterparts. Aneurysms in children are more frequently located in the middle cerebral artery and are often larger, with a significant proportion categorized as giant aneurysms (≥25 mm) (75). The clinical presentation can vary, with many cases presenting with SAH or neurological deficits due to mass effect. Imaging studies, particularly MRA and CTA, play a crucial role in the diagnosis and management of these aneurysms. For instance, MRA has been shown to be effective in identifying the size, location, and morphology of the aneurysms, which are critical for determining the appropriate therapeutic approach (76). Furthermore, the presence of associated systemic diseases, such as sickle cell disease, can significantly influence the imaging features and clinical outcomes of pediatric aneurysms, necessitating a comprehensive evaluation of both the aneurysm and the underlying conditions (77). The management strategies for pediatric aneurysms often differ from adults, with a preference for microsurgical techniques over endovascular approaches, especially in cases involving giant aneurysms (78). As such, understanding the unique imaging characteristics and clinical presentations of pediatric aneurysms is essential for optimizing patient care and outcomes.

Despite their rarity, pediatric aneurysms are associated with a higher risk of rupture and worse outcomes compared to adult aneurysms, underscoring the need for early and accurate detection. Importantly, because of children’s heightened sensitivity to ionizing radiation and the potential need for serial imaging, MRA is generally preferred over CTA in pediatric patients whenever feasible, especially for screening and follow-up (75).

#### 1.7.2 Imaging screening in patients with genetic syndromes

Genetic syndromes such as Loeys-Dietz syndrome and Marfan syndrome are associated with a heightened risk of developing IAs, necessitating tailored imaging screening strategies. These conditions are characterized by connective tissue abnormalities that predispose individuals to vascular complications, including aneurysm formation. For instance, patients with Loeys-Dietz syndrome often present with multiple aneurysms at a young age, highlighting the importance of early and regular imaging surveillance to monitor for aneurysm development and growth (79). Imaging modalities such as MRA and CTA are vital for the detection and characterization of these aneurysms, allowing for timely intervention when necessary. Moreover, the integration of genetic testing into clinical practice can aid in identifying at-risk individuals, thereby facilitating proactive screening measures. For example, in families with a history of aneurysms or those diagnosed with genetic syndromes, routine imaging is recommended to assess for the presence of UIAs (80). This proactive approach not only helps in the early detection of aneurysms but also informs risk assessment and management strategies for affected individuals. Ultimately, the combination of genetic screening and advanced imaging techniques can significantly enhance the monitoring and management of IAs in patients with hereditary conditions, leading to improved clinical outcomes and reduced morbidity associated with aneurysm rupture.

### 1.8 The relationship between imaging data quality and diagnostic accuracy

#### 1.8.1 The impact of imaging acquisition parameters on AI diagnostic performance

The quality of imaging data is paramount in ensuring accurate diagnostic performance, particularly in the context of AI applications in medical imaging. The interplay between radiation dose and image reconstruction techniques significantly influences the quality of images produced, which in turn affects AI diagnostic capabilities. Studies have shown that lower radiation doses can lead to increased image noise and reduced clarity, which may hinder the ability of AI algorithms to detect and classify abnormalities accurately. For instance, research indicates that while iterative reconstruction methods can enhance image quality at lower radiation doses, they may still not achieve the clarity provided by standard-dose images (81). Moreover, the choice of reconstruction algorithms, such as model-based iterative reconstruction (MBIR) versus traditional filtered back projection (FBP), has been shown to impact diagnostic accuracy. MBIR can significantly reduce image noise while maintaining spatial resolution, thus enhancing the visibility of lesions and improving the performance of AI diagnostic tools (82). Therefore, optimizing acquisition parameters, including radiation dose and reconstruction techniques, is critical for maximizing AI diagnostic performance and ensuring that clinicians can rely on these advanced technologies for accurate patient assessments.

Standardization in data acquisition protocols is essential for achieving consistent imaging quality, which directly correlates with diagnostic accuracy. Variability in imaging techniques, such as differences in scanner settings, patient positioning, and the use of contrast agents, can lead to discrepancies in image quality and, consequently, in diagnostic outcomes. For example, a study highlighted that non-standardized imaging protocols could result in significant variations in image quality, affecting the reliability of AI algorithms trained on such data (83). Furthermore, the implementation of standardized protocols not only enhances reproducibility but also facilitates the integration of AI systems into clinical workflows. By ensuring that all imaging data adheres to established standards, healthcare providers can improve the training datasets used for AI algorithms, thereby increasing their robustness and accuracy in clinical applications. In summary, standardization of data acquisition is vital for optimizing image quality and ensuring that AI diagnostic tools can operate effectively and reliably across different clinical settings.

#### 1.8.2 Challenges and opportunities brought by imaging equipment and technology updates

As imaging technology continues to advance, the introduction of new devices and modalities presents both challenges and opportunities regarding data consistency. Different imaging systems may produce varying image qualities and characteristics, which can complicate the integration of AI diagnostic tools that rely on consistent input data. For instance, discrepancies in image resolution, contrast, and noise levels can lead to difficulties in training AI algorithms effectively, as they may not generalize well across diverse imaging datasets (84). This inconsistency can result in reduced diagnostic accuracy when AI systems are applied to images obtained from different devices. To mitigate these challenges, it is essential to establish harmonization protocols that ensure that imaging data from various devices can be standardized or normalized before being utilized for AI training. This approach not only enhances the reliability of AI diagnostics but also promotes interoperability among different imaging systems, ultimately benefiting patient care.

The rapid evolution of imaging technologies necessitates robust imaging quality monitoring and optimization strategies to ensure high diagnostic accuracy. Continuous assessment of imaging quality is critical, particularly with the integration of AI in clinical practice, as these systems depend heavily on the quality of the input data. Implementing regular quality control measures, including routine calibration of imaging devices and assessment of image quality metrics, can help maintain high standards in diagnostic imaging (85). Moreover, the adoption of advanced image processing techniques, such as deep learning-based noise reduction and artifact correction algorithms, can further enhance image quality and mitigate the impact of suboptimal imaging conditions. These strategies not only improve the performance of AI diagnostic tools but also ensure that clinicians can make informed decisions based on accurate and reliable imaging data. Therefore, a proactive approach to imaging quality monitoring and optimization is essential for maximizing the potential of AI in diagnostic imaging.

### 1.9 Future development trends and cutting-edge technology outlook

#### 1.9.1 Multimodal image fusion technology

Multimodal image fusion technology has emerged as a pivotal advancement in the diagnosis and treatment planning of IAs, integrating various imaging modalities such as CTA, MRA, and functional imaging techniques. The fusion of these imaging types allows for a comprehensive assessment of the aneurysm’s size, shape, and relationship to surrounding anatomical structures, which is crucial for surgical planning and intervention. Studies have shown that the use of multimodal imaging significantly enhances the accuracy of aneurysm detection and characterization, leading to improved clinical outcomes. For instance, research indicates that preoperative multimodal imaging can provide neurosurgeons with detailed 3D reconstructions that reflect intraoperative findings, thereby facilitating more precise surgical approaches and reducing complication rates (86). Furthermore, the integration of advanced software tools for image registration and fusion has streamlined the workflow in clinical settings, allowing for real-time visualization of critical anatomical relationships during surgery. As technology progresses, the potential for incorporating AI to automate and enhance the image fusion process is becoming increasingly viable, promising to further improve diagnostic accuracy and treatment efficacy in the management of IAs.

#### 1.9.2 AI and big data-driven personalized diagnosis and treatment

The integration of AI and big data analytics into the field of neurosurgery is revolutionizing personalized diagnosis and treatment strategies for patients with IAs. Beyond isolated image-based predictions, recent advances point toward the value of incorporating clinical features (e.g., age, hypertension, and smoking status), genetic risk factors (e.g., family history, connective tissue disorders), and hemodynamic parameters (e.g., WSS, flow patterns) to construct multidimensional risk profiles. By merging imaging biomarkers with patient-specific data, AI-driven models can improve rupture risk prediction and enable individualized therapeutic decision-making. This multimodal approach aligns with the paradigm of precision medicine and holds the potential to optimize patient outcomes through more targeted and context-aware interventions (9, 49).

#### 1.9.3 Applications of VR and augmented reality in clinical and educational settings

Virtual and augmented reality (VR/AR) technologies are increasingly being explored as tools for enhancing clinical visualization of IAs. By integrating CTA or 3D rotational DSA data into immersive environments, VR systems allow clinicians to interact with patient-specific vascular models in three dimensions, improving spatial understanding of aneurysm morphology and its relationship to surrounding structures. These applications have shown potential in preoperative planning, especially in evaluating clip trajectories or FDs placement (28, 87).

### 1.10 Multidisciplinary collaboration in the comprehensive management of IAs

#### 1.10.1 Collaborative treatment models between radiology and neurosurgery

In the management of IAs, the integration of imaging data into treatment planning is paramount. Advanced imaging modalities, such as DCE-MRI and VWI, provide critical insights into the characteristics of aneurysms, including wall permeability and enhancement, which are associated with rupture risk (6). These imaging techniques allow for a detailed evaluation of aneurysm morphology and hemodynamics, which are essential for selecting appropriate treatment strategies. For instance, the identification of atherosclerotic changes or wall enhancement can influence the decision to opt for surgical clipping versus endovascular coiling (88). Furthermore, the collaboration between radiologists and neurosurgeons facilitates a more nuanced understanding of the aneurysm’s clinical context, enabling tailored treatment plans that consider both anatomical and pathological factors. This multidisciplinary approach not only enhances the precision of interventions but also optimizes patient outcomes by ensuring that the chosen treatment aligns with the specific risk profile of the aneurysm.

The collaborative framework between radiology and neurosurgery extends beyond initial treatment planning to encompass comprehensive preoperative assessments and postoperative follow-ups. Preoperatively, imaging specialists play a crucial role in providing detailed anatomical maps and functional assessments that inform surgical strategies. For example, high-resolution imaging can reveal crucial details about the aneurysm’s relationship with surrounding neurovascular structures, which is vital for surgical planning (4). Postoperatively, the use of advanced imaging techniques for follow-up allows for the early detection of complications such as recanalization or new aneurysm formation, enabling timely interventions (89). This continuous feedback loop between imaging and clinical practice fosters a culture of proactive management, where both radiologists and neurosurgeons work in tandem to monitor patient progress and adapt treatment plans as necessary. Such collaborative mechanisms not only improve patient safety but also enhance the overall efficacy of aneurysm management strategies.

#### 1.10.2 Development of clinical decision support systems

The advent of clinical decision support systems (CDSS) represents a significant advancement in the management of IAs, particularly through the integration of imaging data with clinical information. These intelligent systems leverage AI algorithms to analyze complex datasets, including imaging findings and patient demographics, to assist clinicians in making informed decisions (46). For instance, AI-driven models can predict the likelihood of aneurysm rupture based on imaging characteristics and patient risk factors, thereby guiding treatment options and surveillance strategies. Moreover, the incorporation of machine learning techniques into CDSS enhances diagnostic accuracy and helps in stratifying patients based on their individual risk profiles (90). Such systems not only streamline the decision-making process but also reduce variability in clinical practice, ensuring that patients receive evidence-based care tailored to their specific needs.

The implementation of AI-assisted CDSS has the potential to significantly improve diagnostic efficiency and patient prognosis in the context of IAs. By automating the analysis of imaging data, these systems can quickly identify critical features that may be indicative of aneurysm instability or growth, allowing for timely interventions (91). Furthermore, predictive analytics can provide clinicians with insights into potential outcomes based on historical data, thereby facilitating proactive management strategies. For example, a system that integrates imaging data with clinical outcomes can help identify patients who are at higher risk for complications, thereby guiding more intensive monitoring or earlier surgical intervention (92). As these systems evolve, they hold the promise of transforming the landscape of aneurysm management, enabling a more personalized approach that not only enhances patient safety but also optimizes overall treatment efficacy.

## 2 Conclusion

In conclusion, the evolution of imaging techniques for IAs diagnosis has witnessed remarkable advancements, transitioning from two-dimensional representations to sophisticated 3D models, and from manual interpretations to intelligent-assisted methodologies. This progression has significantly enhanced the accuracy and efficiency of diagnosing these critical vascular anomalies. As the cornerstone imaging modalities—CTA, MRA, and DSA—continue to play a pivotal role in clinical diagnostics, the integration of 3D reconstruction and hemodynamic analysis has provided invaluable support for risk assessment, enabling clinicians to make more informed decisions regarding patient management (93).

The introduction of AI into the realm of aneurysm detection and risk prediction represents a transformative leap forward (46). AI technologies have the potential to automate detection and segmentation processes, thus streamlining workflows and reducing human error. However, challenges remain, particularly concerning data quality and the identification of small aneurysms, which necessitate ongoing research and development. Addressing these challenges is critical to fully realize the potential of AI in clinical practice.

Moreover, imaging plays an indispensable role not only in diagnosis but also in treatment planning, surgical navigation, and postoperative follow-up (94). The integration of imaging modalities fosters multidisciplinary collaboration, allowing for a more comprehensive approach to patient care. This collaborative framework enhances communication among specialists, ultimately leading to improved patient outcomes.

Looking ahead, the future of imaging in IAs diagnosis and management appears promising, especially with the anticipated advancements in multimodal imaging fusion, VR technologies, and big data analytics (95). These innovations are expected to facilitate more precise and personalized approaches to patient care, thereby elevating the quality of medical services provided to individuals affected by IAs.

In summary, the landscape of IAs imaging is evolving rapidly, driven by technological advancements and collaborative efforts. As we continue to navigate the complexities of this field, it is crucial to balance various research perspectives and findings, ensuring that we remain focused on the ultimate goal: delivering superior healthcare outcomes for patients with IAs. The journey ahead is filled with opportunities, and by embracing innovation and collaboration, we can significantly enhance the diagnostic and therapeutic landscape for these potentially life-threatening conditions.

## 3 Limitations

Despite our effort to provide a comprehensive review, this study has several limitations. First, although we included a broad range of literature on imaging techniques, AI applications, and hemodynamic modeling, the rapid evolution of these technologies may render some information outdated in the near future. Second, while we attempted to balance coverage across clinical, technical, and computational perspectives, some emerging areas such as molecular imaging or ultra-high-field MRI were not discussed in depth. Additionally, given the inherent variability in imaging protocols and institutional practices, the generalizability of some cited findings may be limited. Lastly, as with any narrative review, there may be selection bias in the literature included.
